# Hypercalcemic Crisis: A Rare Presentation of Ewing’s Sarcoma

**DOI:** 10.7759/cureus.50339

**Published:** 2023-12-11

**Authors:** Abhijeet Haldhar, Rajshekhar Lohar, Saurabh S Kumbhar, Ravi Kant, Minakshi Dhar

**Affiliations:** 1 Internal Medicine, All India Institute of Medical Sciences, Rishikesh, Rishikesh, IND; 2 College of Nursing, All India Institute of Medical Sciences, Rishikesh, Rishikesh, IND; 3 Geriatric Medicine, All India Institute of Medical Sciences, Rishikesh, Rishikesh, IND

**Keywords:** primitive neuroectodermal tumors, ewing’s sarcoma, multifocal ewing sarcoma, small round blue cell tumor, ewing sarcoma family of tumors (esft), hypercalcemic crisis, severe hypercalcemia, ewing

## Abstract

Ewing’s sarcoma (ES) is a rare malignancy of adolescence that usually presents with clinically apparent disease involving long bones, brought to attention by trauma and/or fractures. Hypercalcemia of malignancy is a well-known phenomenon; however, hypercalcemia is exceptionally rare in ES. This case report discusses a 20-year-old lady who experienced chronic bone pains in her hip and lower limbs, ultimately leading to a hypercalcemic crisis. We emphasize the importance of considering ES as a potential cause of hypercalcemia, highlighting the mechanism, diagnostic and therapeutic challenges, the associated poor prognosis, and the necessity for a multidisciplinary approach to managing the condition.

## Introduction

Ewing’s sarcoma (ES) previously known as Ewing sarcoma family tumor (ESFT) or primitive neuroectodermal tumor, is a rare malignancy, first described by James Ewing in 1921 as an undifferentiated primary bone tumor involving the diaphysis of long bones [1]. It is characterized by morphologically similar small round blue cell neoplasm and a common chromosomal translocation, t(11;22)(q24;q12). Globally, incidence per million of ES varies according to ethnicity and region, with the highest incidence seen in Spain (3.5) and the lowest in China (0.5); India stands in between with an incidence of 1.5 to 2 [2].

ES commonly affects young children and adolescents. Often patients present with clinically apparent localized disease involving long bones and the pelvis, brought to attention by pathological fractures. However, some may have occult metastatic disease and dissemination. Presentation of ES with hypercalcemia is even rarer. ESFT group of tumors includes ES, peripheral primitive neuroectodermal tumor, rhabdomyosarcoma synovial sarcoma, non-Hodgkin’s lymphoma, retinoblastoma, neuroblastoma, hepatoblastoma, and nephroblastoma or Wilms’ tumor. As these tumors share a similar morphology, differentiation between them is aided by immunohistochemical markers. Multidrug chemotherapy as well as local disease control with surgery and/or radiation therapy are indicated for nearly all patients.

## Case presentation

A 20-year-old female hailing from the sub-Himalayan region of India and denying any comorbidities presented with complaints of insidious-onset progressively worsening hip pain and bilateral lower limb pain for the last three years, which aggravated on movement and relieved by over-the-counter analgesics. Over time, she developed excruciating pain resulting in a bedridden state for the last two weeks, and became dependent on family members for activities of daily living. In the last two days, she developed restlessness which was followed by a decreased level of consciousness. She had no history of trauma, fever, nausea, vomiting, seizures, radiation of pain, lower limb weakness, loss of sensations, or bowel or bladder involvement. There was no family history of suggestive malignancy and tuberculosis.

On examination, she was dehydrated, agitated, and confused; neurological examination showed no signs of meningeal irritation or focal neurological deficits. Other systemic examinations were unremarkable. ECG tracing showed sinus tachycardia with a short QT segment, and point-of-care echocardiography showed normal contractility and left ventricular ejection fraction (~60%) and collapsible inferior vena cava. A complete hemogram showed mild anemia and neutrophilic leukocytosis. Liver function tests showed elevated alkaline phosphatase (ALP) and serum lactate dehydrogenase (LDH). Renal biochemistry showed hypercalcemia and pre-renal type of acute kidney injury (AKI) (Table 1). Cerebrospinal fluid (CSF) analysis and computed tomography (CT) of the head were unremarkable. An infective panel comprising workup for tuberculosis and blood and urine cultures were negative, and routine abdominal had no abnormality. To note, she also had markedly elevated inflammatory markers.

**Table 1:** Investigation chart. Note, elevated blood urea, serum creatinine, and raised serum calcium levels which improved with appropriate therapy over the course. Also, note elevated ALP levels secondary to osteolysis, and marked elevation of inflammatory markers ESR, CRP, and serum ferritin; in contrast, decreased serum albumin (negative acute-phase reactant) can also be seen. In addition, the patient had anemia and raised LDH levels, which are poor prognostic markers of the disease. It can be noted that the patient had normal levels of PTH. Unfortunately, parathyroid hormone-related protein could not be done. ALP: alkaline phosphatase; LDH: lactate dehydrogenase; PTH: parathyroid hormone; ESR: erythrocyte sedimentation rate; CRP, C-reactive protein

Investigation (units)	Day 1	Day 5	Day 9	Day 13	Day 30	Day 60	Reference range
Blood urea (mg/dL)	75	36	21	12	14	10	10–43
Serum creatinine (mg/dL)	2.76	1.62	0.95	0.51	0.27	0.22	0.5–1.0
Serum alcium (mg/dL)	14.7	11.3	7.8	6.9	7.4	8.6	8.0–10.8
Serum protein (g/dL)	6.0	4.2	4.3	4.5	5.7	5.8	6.40–8.30
Serum albumin (g/dL)	3.2	2.0	2.1	2.0	2.3	2.6	3.50–5.00
Serum ALP (U/L)	1,950	1,554	1,445	1,206	1,913	948	Upto 270
Serum LDH (U/L)			1,873.5		1,599	402	240–480
Serum PTH (pg/mL)	23.6						15–68
Vitamin D (ng/mL)	10.80						30–50
Hemoglobin (g/dL)	10.03	6.1	7.5	7.0	8.3	8.9	12–15
Total leukocyte count (thousand/mm^3^)	13.66	4.7	6.6	9.4	10.0	13.7	4–11
Platelets (thousand/mm^3^)	179	152	183	172	375	386	150–400
ESR (mm, in the first hour)		85					0–20
CRP (mg/L)		150.5					0–1.0
Serum ferritin (ng/mL)		3922					10–291

**Figure 1:**
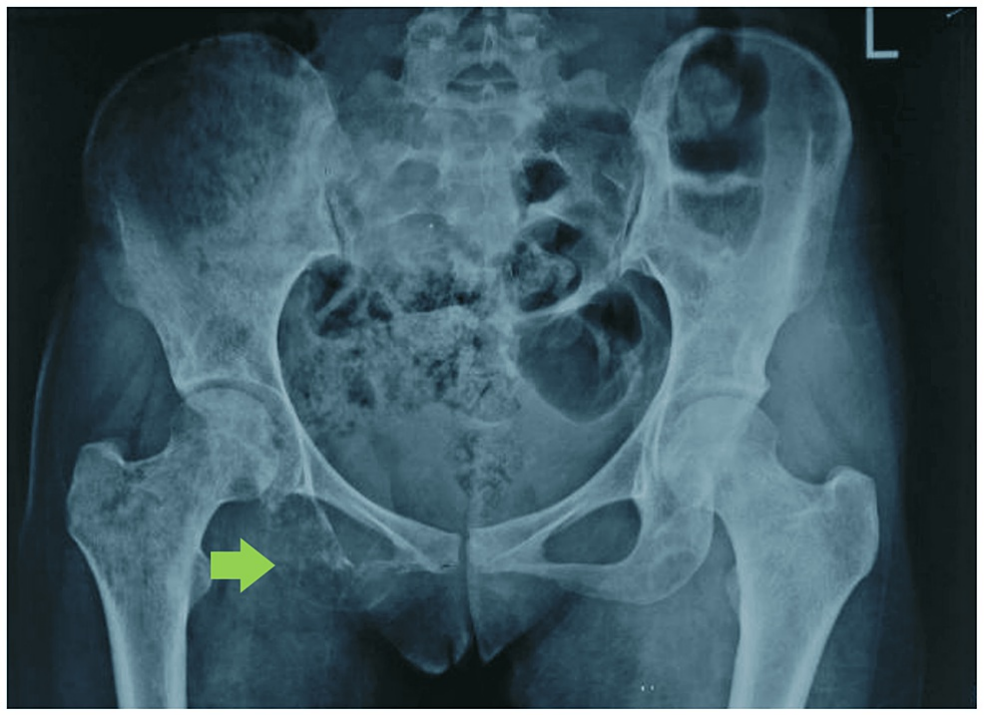
Pelvic radiograph showing primary osteolytic lesions in right ischial tuberosity (green arrow).

**Figure 2:**
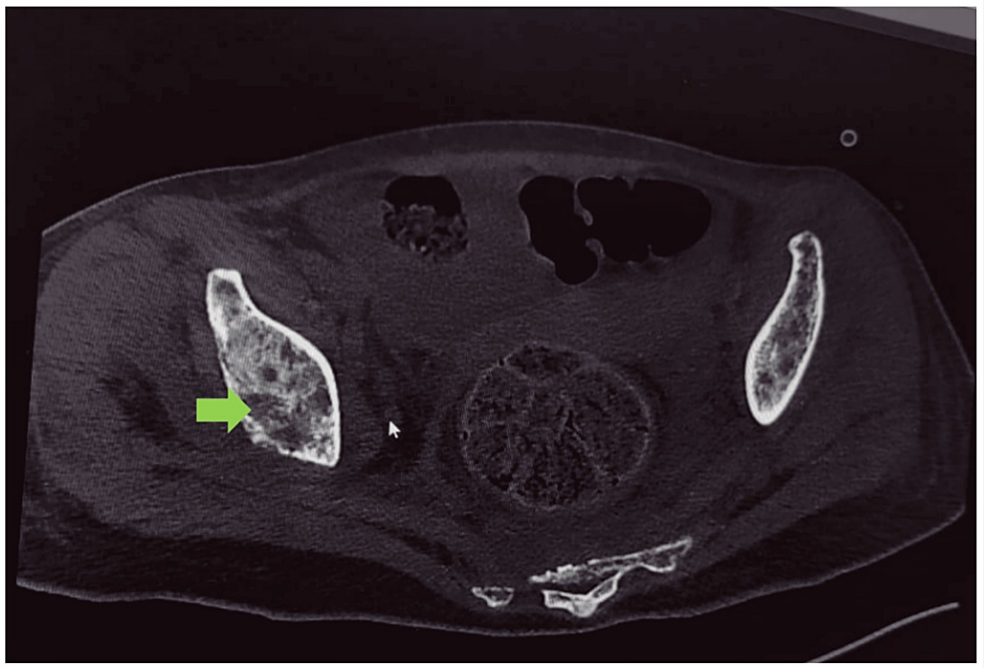
Non-contrast computed tomography showing multiple lytic lesions with aggressive periosteal reaction and a large soft tissue component epicenter in the right ischium (green arrow).

**Figure 3:**
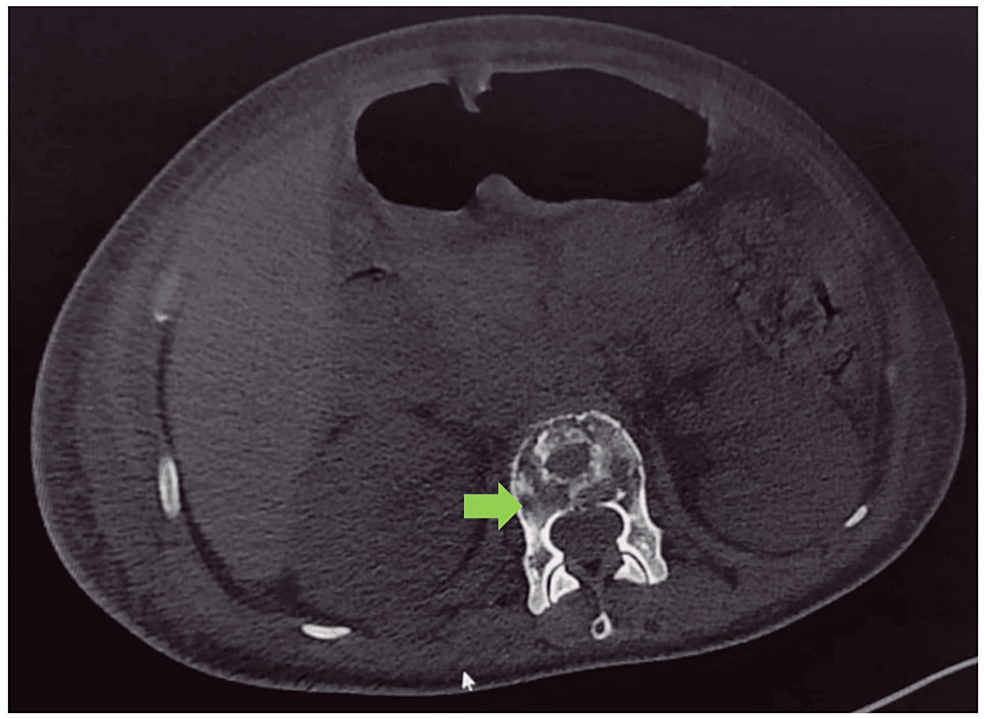
Non-contrast computed tomography showing numerous lytic lesions thoracic vertebral body (green arrow).

Altered sensorium and deranged renal functions were attributed to the hypercalcemic crisis. She was managed with aggressive fluid therapy, diligent monitoring, intravenous bisphosphonates, and calcitonin therapy. This was followed by improvement in her sensorium, and renal functions returned to normal. Simultaneously, causes of hypercalcemia were evaluated. Ultrasonography of the thyroid gland, thyroid function, and parathormone levels were within normal limits. However, parathyroid hormone-related protein (PTHrP) was not tested due to the non-availability of the test in the institute and financial constraints. Excruciating bony pains were treated with opioid analgesics. In suspicion of malignancy-associated hypercalcemia, a skeletal survey was ordered which revealed multiple lytic lesions and suspected primary lesions in the pelvis (Figure 1). Further, CT imaging revealed lytic lesions with aggressive periosteal reaction and a large soft tissue component epicenter in the right ischium along with numerous lytic lesions involving the axial and appendicular skeleton (Figures 2, 3).

**Figure 4:**
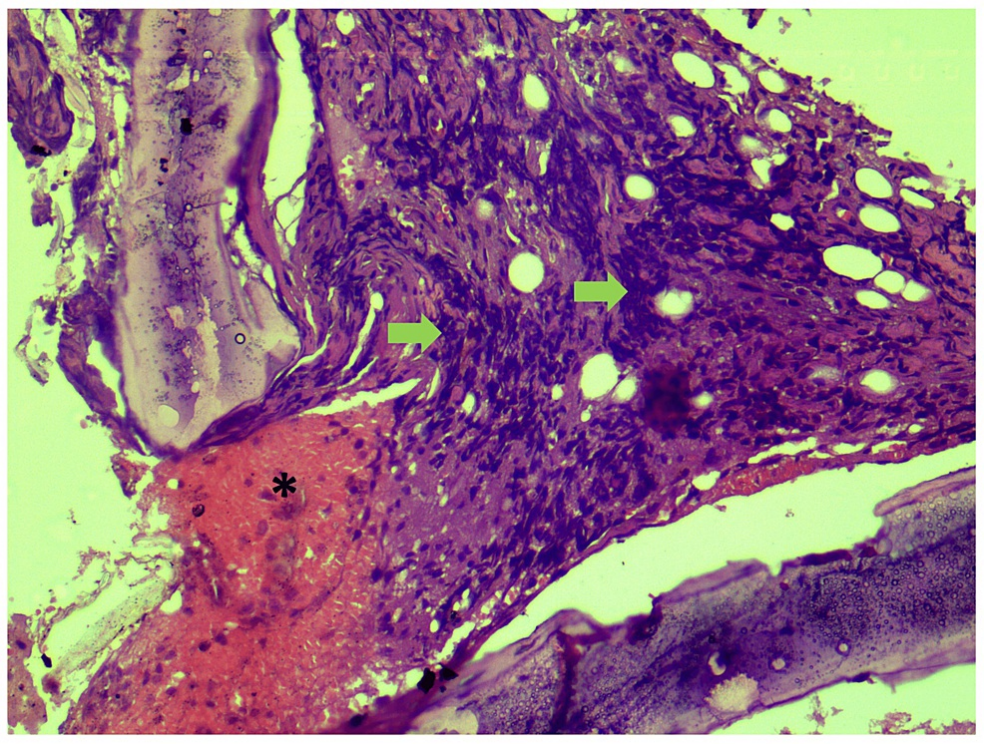
Histopathological section (hematoxylin and eosin staining) of the specimen showing infiltrating small round blue cells (green arrows) with large areas of hemorrhage (asterisk), bony trabeculae, and intervening marrow elements.

**Figure 5:**
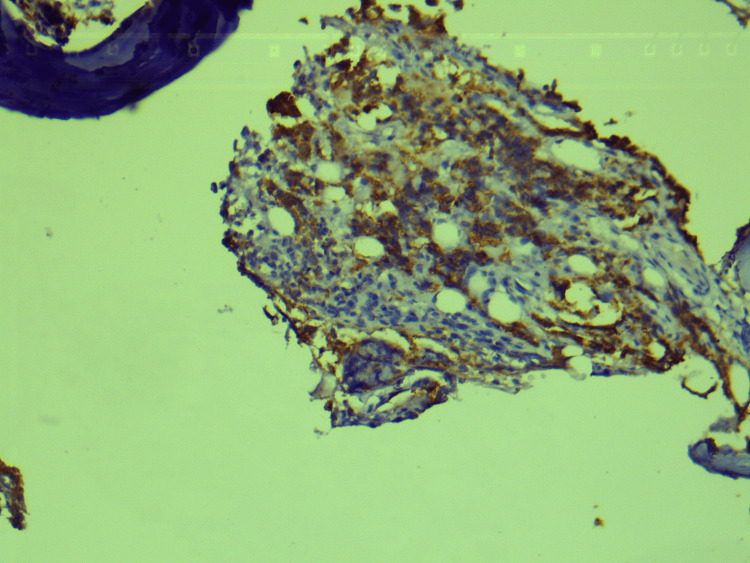
Immunohistochemistry: positive for CD99.

**Figure 6:**
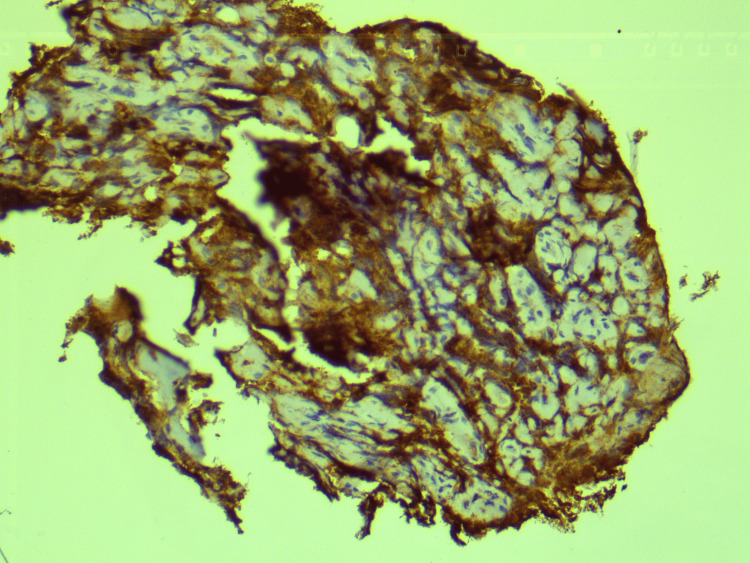
Immunohistochemistry demonstrating positivity for neuron-specific enolase.

**Figure 7:**
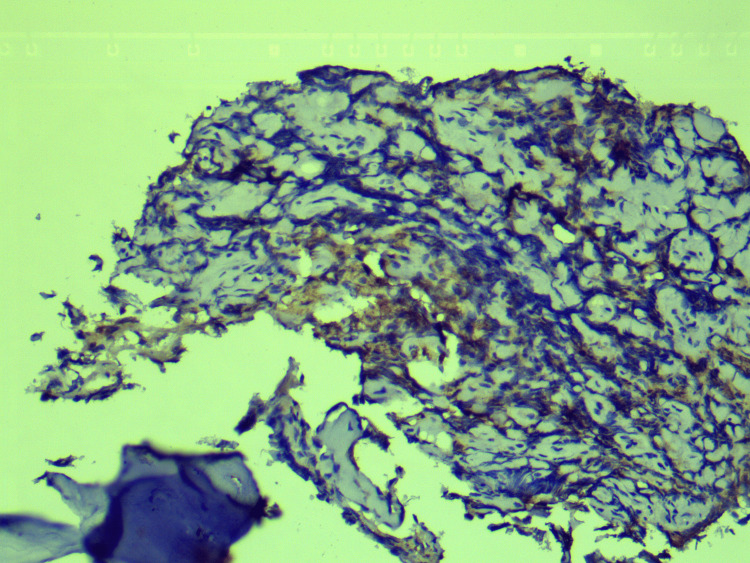
Immunohistochemistry showing positivity for synaptophysin.

**Figure 8:**
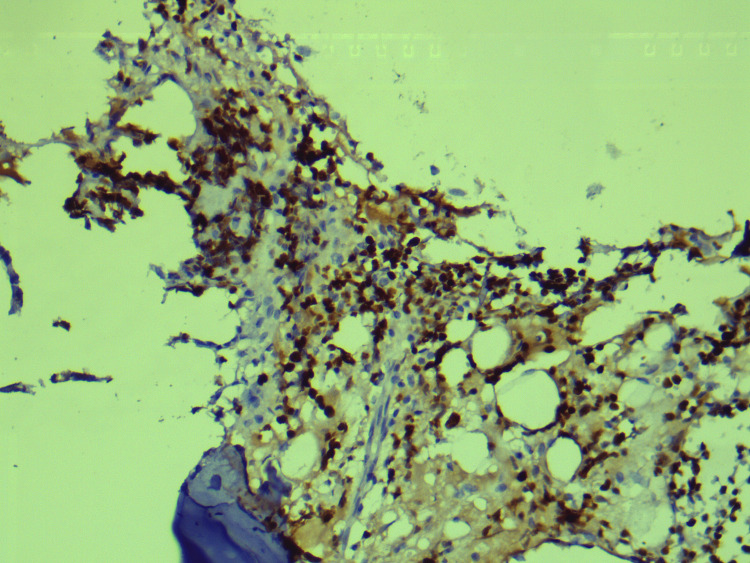
Immunohistochemistry: Ki-67, 70-80%.

Histopathological examination of CT-guided biopsy of the lesion revealed large areas of hemorrhage, bony trabeculae, intervening marrow elements, and infiltration by small round blue cells (Figure 4). This was further subjected to immunohistochemistry which revealed positivity for CD99, neuron-specific enolase, and CD36. It also revealed focal positivity for synaptophysin, and Ki-67 was 70-80%, confirming a diagnosis of ES (Figures 5-8).

She was following up in medical oncology for chemotherapy and palliative radiotherapy on an outpatient/daycare basis. She had a slight improvement in her general conditions for a brief period. However, she did not tolerate further chemotherapy and deteriorated further after the fifth cycle of chemotherapy. She did not receive the sixth cycle of chemotherapy and was lost to follow-up. On enquiring further, it was noted that she succumbed to her illness a few months later.

## Discussion

Hypercalcemia is the leading cause of paraneoplastic syndrome. However, the most common cause of asymptomatic hypercalcemia is benign parathyroid adenoma or hyperplasia. Symptomatic hypercalcemia is usually associated with underlying malignancy, usually squamous cell lung carcinoma. About 20-30% of cancer patients develop hypercalcemia during illness, and this condition is strongly associated with a grave prognosis, merely half of them survive a month [3].

In contrast, hypercalcemia occurring in ES is exceptionally rare, with only a few documented instances. Notable reports include those by Lozance et al. (1994) [4] and Kurihashi et al. (1996) [5], where hypercalcemia was observed in cases of ES with disseminated disease. A more recent report by Roy et al. [6] in 2022 documented a hypercalcemic crisis in a non-metastatic ES of the kidney.

Hypercalcemia in the context of cancer can be attributed to four distinct mechanisms. Local osteolytic hypercalcemia occurs due to a substantial increase in osteoclastic bone resorption in regions surrounding malignant cells within the bone marrow. Humoral hypercalcemia of malignancy arises from the systemic secretion of PTHrP by the cancerous tumors, leading to elevated calcium levels. Some cancers can secrete the active form of vitamin D, known as 1,25-dihydroxyvitamin D, contributing to hypercalcemia. Finally, a very rare occurrence involves the ectopic secretion of parathyroid hormone, a phenomenon observed in only a limited number of reported cases [3].

AKI associated with hypercalcemia develops due to direct renal vasoconstriction and volume depletion. Volume depletion results from various mechanisms, including downregulation of aquaporin-2 channels, tubulointerstitial injury by calcium deposition in the medulla, activation of the calcium-sensing receptors on the basolateral membrane of the thick ascending limb of the loop of Henle, Na^+^/K^+^/2Cl^-^ channel inhibition, and PGE2 generation resulting in reduced sodium chloride reabsorption in the loop of Henle which also aids to hypovolemia. This is usually reversible by volume expansion [7].

Adequate hydration is the cornerstone of treatment; asymptomatic or mildly symptomatic hypercalcemia is treated with oral or intravenous fluid therapy. Moderate-to-severe hypercalcemia is treated with more aggressive intravenous fluid therapy with or without diuretics. In a hypercalcemic crisis, without peripheral edema and AKI, normal saline is to be initiated at a rate of 200 to 300 mL/hour which can be adjusted to maintain the urine output at 100 to 150 mL/hour. Bisphosphonates and calcitonin are usually required in severe cases along with hydration. However, for patients with refractory hypercalcemia, heart failure, AKI, and peripheral edema renal replacement therapy is indicated.

Metastasis most commonly occurs in the lungs, followed by the bone and bone marrow in ES. Isolated metastasis to the lungs generally leads to a more favorable prognosis compared to bone involvement. ES is characterized by its propensity to affect multiple bones and bone-to-bone metastasis. This bone-to-bone metastatic pattern, along with marrow involvement and osteolysis, contributes to the development of hypercalcemia through increased bone resorption, subsequently leading to kidney injury. Patients invariably experience bone pain and are at risk of developing pathological fractures [8].

Initial laboratory assessments should encompass a complete blood count, serum chemistry analysis, and the measurement of lactate dehydrogenase levels. LDH is a recognized prognostic factor in individuals diagnosed with ES. Furthermore, to establish a diagnosis, it is crucial to eliminate other potential causes of hypercalcemia. It's important to bear in mind the necessity of conducting a comprehensive skeletal examination in these patients to identify osteolytic lesions as a significant contributor to hypercalcemia. The presence of bone lytic lesions resulting from distant metastases, which frequently affect vertebral bodies, is a common occurrence in neoplastic conditions such as breast and prostate carcinoma. This presentation is a telltale sign of certain diseases such as multiple myeloma and various plasma cell disorders, necessitating a bone marrow examination [8]. An image-guided biopsy should be performed on suspicious or primary lesions for histopathological and immunohistochemical analysis. The diagnostic evaluation for metastasis and staging involves the use of diagnostic imaging techniques such as CT, magnetic resonance imaging, positron emission tomography, and radionuclide bone scans.

The prognosis of ES is influenced by several key factors. The extent of the disease plays a significant role, with metastatic cases at diagnosis having a poorer outcome, resulting in a 70% survival rate for localized cases compared to 33% for non-metastatic ones. Tumor size and location also matter, with tumors in the axial region and larger primary tumors having worse outcomes. Laboratory findings such as fever, anemia, and elevated LDH levels are linked to larger disease volume and poorer prognosis. Response to treatment is crucial, with chemotherapy significantly improving outcomes. Histology, older age, and molecular findings also impact prognosis. Molecular diversity once influenced prognosis but is less significant with modern treatments [8,9].

## Conclusions

This case itself is unique - “ES presenting as a hypercalcemic crisis.” Hypercalcemia in ES is exceptionally rare. It is primarily linked to bone-to-bone metastasis and osteolysis, called local osteolytic hypercalcemia; in contrast to more common PTHrP-associated hypercalcemia of malignancy. Nevertheless, management of such patients remains aggressive hydration with normal saline, intravenous bisphosphonates, and calcitonin. These patients require diligent monitoring of intake and output. While most patients recover, some might develop AKI or refractory hypercalcemia, needing renal replacement therapy. Perhaps, the prognosis of hypercalcemia even in crisis is good. However, the prognosis in ES presenting with hypercalcemia or hypercalcemic crisis is grave. It points toward disseminated or multifocal disease which in itself has poor prognosis. The overall prognosis of ES is multifactorial, influenced by disease extent, tumor characteristics, tumor burden, and response to treatment. Clinical findings such as older age at presentation rather than adolescents, excruciating multifocal pain, weight loss, and fever make the prognosis worse. Laboratory parameters such as anemia, elevated LDH levels, and raised inflammatory markers are associated with poor prognosis. In addition, axial involvement (especially pelvic disease) and multifocal or disseminated disease are associated with poor prognosis and were noted in this patient. Molecular diversity once thought to influence prognosis is less significant with modern therapy. This concise overview underscores the intricate nature and poor prognosis of ES, especially with hypercalcemia, emphasizing the need for comprehensive clinical evaluation, multidisciplinary approach, aggressive management, and tailored therapeutic approaches.
